# Global Initiative for Children’s Surgery: A Model of Global Collaboration to Advance the Surgical Care of Children

**DOI:** 10.1007/s00268-018-04887-8

**Published:** 2019-01-08

**Authors:** Global Initiative for Children’s Surgery, Naomi Wright, Guy Jensen, Etienne St-Louis, David Grabski, Yasmine Yousef, Neema Kaseje, Laura Goodman, Jamie Anderson, Emmanuel Ameh, Tahmina Banu, Stephen Bickler, Marilyn Butler, Michael Cooper, Zipporah Gathuya, Patrick Kamalo, Bertille Ki, Rashmi Kumar, Vrisha Madhuri, Keith Oldham, Doruk Ozgediz, Dan Poenaru, John Sekabira, Lily Saldaña Gallo, Sabina Siddiqui, Benjamin Yapo, Francis A. Abantanga, Mohamed Abdelmalak, Nurudeen Abdulraheem, Niyi Ade-Ajayi, Edna Adan Ismail, Adesoji Ademuyiwa, Eltayeb Ahmed, Sunday Ajike, Olugbemi Benedict Akintububo, Felix Alakaloko, Brendan Allen, Vanda Amado, Shanthi Anbuselvan, Theophilus Teddy Kojo Anyomih, Leopold Asakpa, Gudeta Assegie, Jason Axt, Ruben Ayala, Frehun Ayele, Harshjeet Singh Bal, Rouma Bankole, Tim Beacon, Zaitun Bokhari, Hiranya Kumar Borah, Eric Borgstein, Nick Boyd, Jason Brill, Britta Budde-Schwartzman, Fred Bulamba, Bruce Bvulani, Sarah Cairo, Juan Francisco Campos Rodezno, Massimo Caputo, Milind Chitnis, Maija Cheung, Bruno Cigliano, Damian Clarke, Tessa Concepcion, Scott Corlew, David Cunningham, Sergio D’Agostino, Shukri Dahir, Bailey Deal, Miliard Derbew, Sushil Dhungel, David Drake, Elizabeth Drum, Bassey Edem, Stella Eguma, Olumide Elebute, Beda R. Espineda, Samuel Espinoza, Faye Evans, Omolara Faboya, Jacques Fadhili Bake, Tatiana Fazecas, Mohammad Rafi Fazli, Graham Fieggen, Anthony Figaji, Jean Louis Fils, Tamara Fitzgerald, Randall Flick, Gacelle Fossi, George Galiwango, Mike Ganey, Maryam Ghavami Adel, Vafa Ghorban Sabagh, Sridhar Gibikote, Hetal Gohil, Sarah Greenberg, Russell Gruen, Lars Hagander, Rahimullah Hamid, Erik Hansen, William Harkness, Mauricio Herrera, Intisar Hisham, Andrew Hodges, Sarah Hodges, Ai Xuan Holterman, Andrew Howard, Romeo Ignacio, Dawn Ireland, Enas Ismail, Rebecca Jacob, Anette Jacobsen, Zahra Jaffry, Deeptiman James, Ebor Jacob James, Adiyasuren Jamiyanjav, Kathy Jenkins, Maria Jimenez, Tarun John K Jacob, Walter Johnson, Anita Joselyn, Nasser Kakembo, Phyllis Kisa, Peter Kim, Krishna Kumar, Charlotte Kvasnovsky, Ananda Lamahewage, Monica Langer, Christopher Lavy, Taiwo Lawal, Colin Lazarus, Andrew Leather, Chelsea Lee, Basil Leodoro, Allison Linden, Katrine Lofberg, Jonathan Lord, Jerome Loveland, Leecarlo Millano Lumban Gaol, Pavrette Magdala, Luc Kalisya Malemo, Aeesha Malik, John Mathai, Marcia Matias, Bothwell Mbuwayesango, Merrill McHoney, Liz McLeod, Ashish Minocha, Charles Mock, Mubarak Mohamed, Ivan Molina, Ashika Morar, Zahid Mukhtar, Mulewa Mulenga, Bhargava Mullapudi, Jack Mulu, Byambajav Munkhjargal, Arlene Muzira, Mary Nabukenya, Mark Newton, Jessica Ng, Karissa Nguyen, Laurence Isaaya Ntawunga, Peter M. Nthumba, Alp Numanoglu, Benedict Nwomeh, Kristin Ojomo, Maryrose Osazuwa, Emmanuel Owusu Abem, Shazia Peer, Norgrove Penny, Robin Petroze, Vithya Priya, Ekta Rai, Lola Raji, Vinitha Paul Ravindran, Desigen Reddy, Henry Rice, Yona Ringo, Amezene Robelie, Jose Roberto Baratella, David Rothstein, Coleen Sabatini, Soumitra Saha, Saurabh Saluja, Lubna Samad, Justina Seyi-Olajide, Bello B. Shehu, Ritesh Shrestha, David Sigalet, Martin Situma, Emily Smith, Adrienne Socci, David Spiegel, Peter Ssenyonga, Jacob Stephenson, Erin Stieber, Richard Stewart, Vinayak Shukla, Thomas Sims, Faustin Felicien Mouafo Tambo, Robert Tamburro, Mansi Tara, Ahmad Tariq, Reju Thomas, Leopold Torres Contreras, Stephen Ttendo, Benno Ure, Luca Vricella, Luis Vasquez, Vijayakumar Raju, Jorge Villacis, Gustavo Villanova, Catherine deVries, Amira Waheeb, Saber Waheeb, Albert Wandaogo, Anne Wesonga, Omolara Williams, Sigal Willner, Nyo Nyo Win, Hussein Wissanji, Paul Mwindekuma Wondoh, Garreth Wood, George Youngson, Denléwendé Sylvain Zabsonre, Luis Enrique Zea Salazar, Adiyasuren Zevee, Bistra Zheleva, Kokila Lakhoo, Diana Farmer

**Affiliations:** King’s Centre for Global Health and Health Partnerships, School for Population Health and Environmental Sciences, King’s College London, Denmark Hill Campus, Suite 2.13, Weston Education Centre, Cutcombe Road, London, SE5 9RJ UK

## Abstract

**Background:**

Recommendations by the Lancet Commission on Global Surgery regarding surgical care in low- and middle-income countries (LMICs) require development to address the needs of children. The Global Initiative for Children’s Surgery (GICS) was founded in 2016 to identify solutions to problems in children’s surgery by utilizing the expertise of practitioners from around the world. This report details this unique process and underlying principles.

**Methods:**

Three global meetings convened providers of surgical services for children. Through working group meetings, participants reviewed the status of global children’s surgery to develop priorities and identify necessary resources for implementation. Working groups were formed under LMIC leadership to address specific priorities. By creating networking opportunities, GICS has promoted the development of LMIC-LMIC and HIC-LMIC partnerships.

**Results:**

GICS members identified priorities for children’s surgical care within four pillars: infrastructure, service delivery, training and research. Guidelines for provision of care at every healthcare level based on these pillars were created. Seventeen subspecialty, LMIC chaired working groups developed the Optimal Resources for Children’s Surgery (OReCS) document. The guidelines are stratified by subspecialty and level of health care: primary health center, first-, second- and third-level hospitals, and the national children’s hospital. The OReCS document delineates the personnel, equipment, facilities, procedures, training, research and quality improvement components at all levels of care.

**Conclusion:**

Worldwide collaboration with leadership by providers from LMICs holds the promise of improving children’s surgical care. GICS will continue to evolve in order to achieve the vision of safe, affordable, timely surgical care for all children.

## Defining the need

### Surgical burden of disease

Since the conclusion of the United Nations’ Millennium Development Goals (MDGs) and through the ongoing work toward Sustainable Development Goals (SDGs), tremendous progress has been made toward reducing childhood mortality [1, 2]. However, the care of children with surgical diseases remains an underappreciated and underfunded area in health care, despite congenital anomalies making up 9% of the surgical burden of disease worldwide, of which two-thirds may be avertable with surgical intervention [3, 4]. Trauma, abdominal emergencies and tumors also contribute to the burden of surgical disease for patients of all ages [5, 6]. In a world where estimates attribute one third of all childhood deaths to a surgical condition, it is unrealistic to believe that we will achieve the SDG 3.2 to end preventable deaths of newborns and children under the age of five by 2030 without real investment in the realm of children’s surgery [2, 7].

### Surgery is (still) not a priority in global health

Publication of the Lancet Commission on Global Surgery as well as a surgical volume within the Disease Control Priorities 3 has raised the profile of global surgery in the global health realm [5, 8]. Surgical care is a cost-effective means for improving childhood health outcomes [4, 5, 9–11]. However, the role of surgery and anesthesia in the first and third SDGs (reducing poverty and improving health and well-being) is not mentioned in the 2030 Agenda for Sustainable Development [2]. World Health Assembly resolution WHA68.15 finally recognized the importance of strengthening emergency and essential surgical care as a component of universal health care [12].

**Fig. 1:**
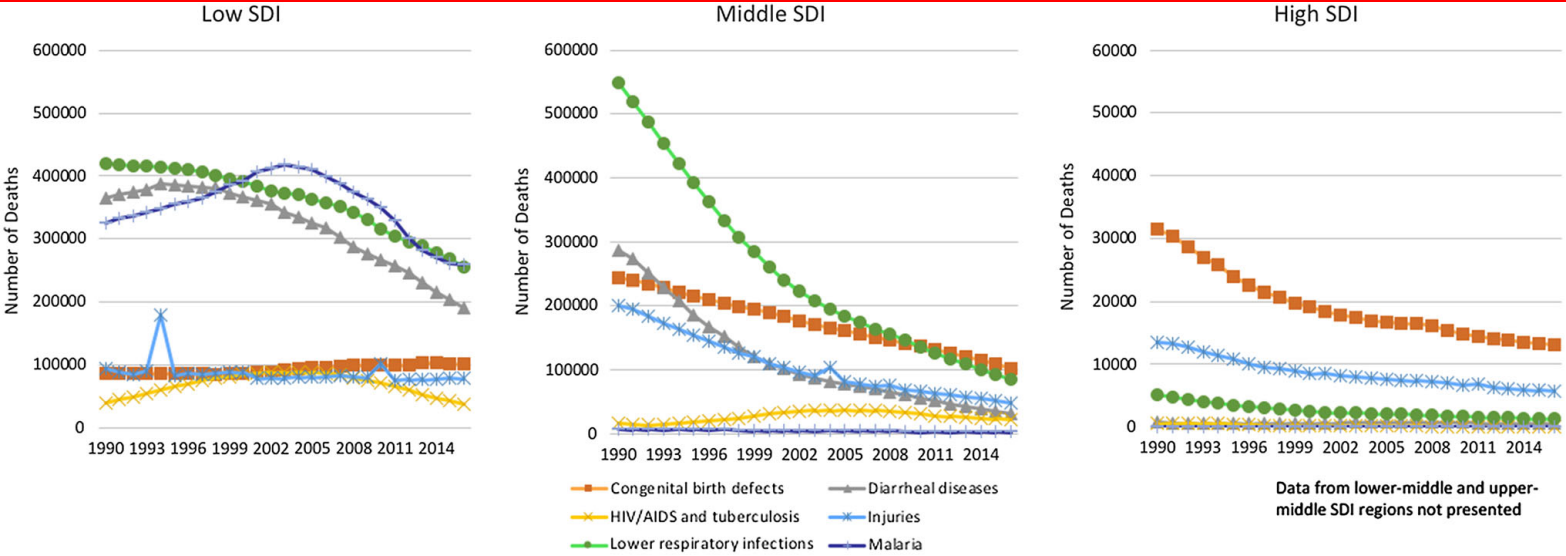
Change in etiology of deaths in children < 5 years of age in low-, middle- and high Socio-Demographic Index (SDI) countries from 1990 to 2016 [13]

**Fig. 2:**
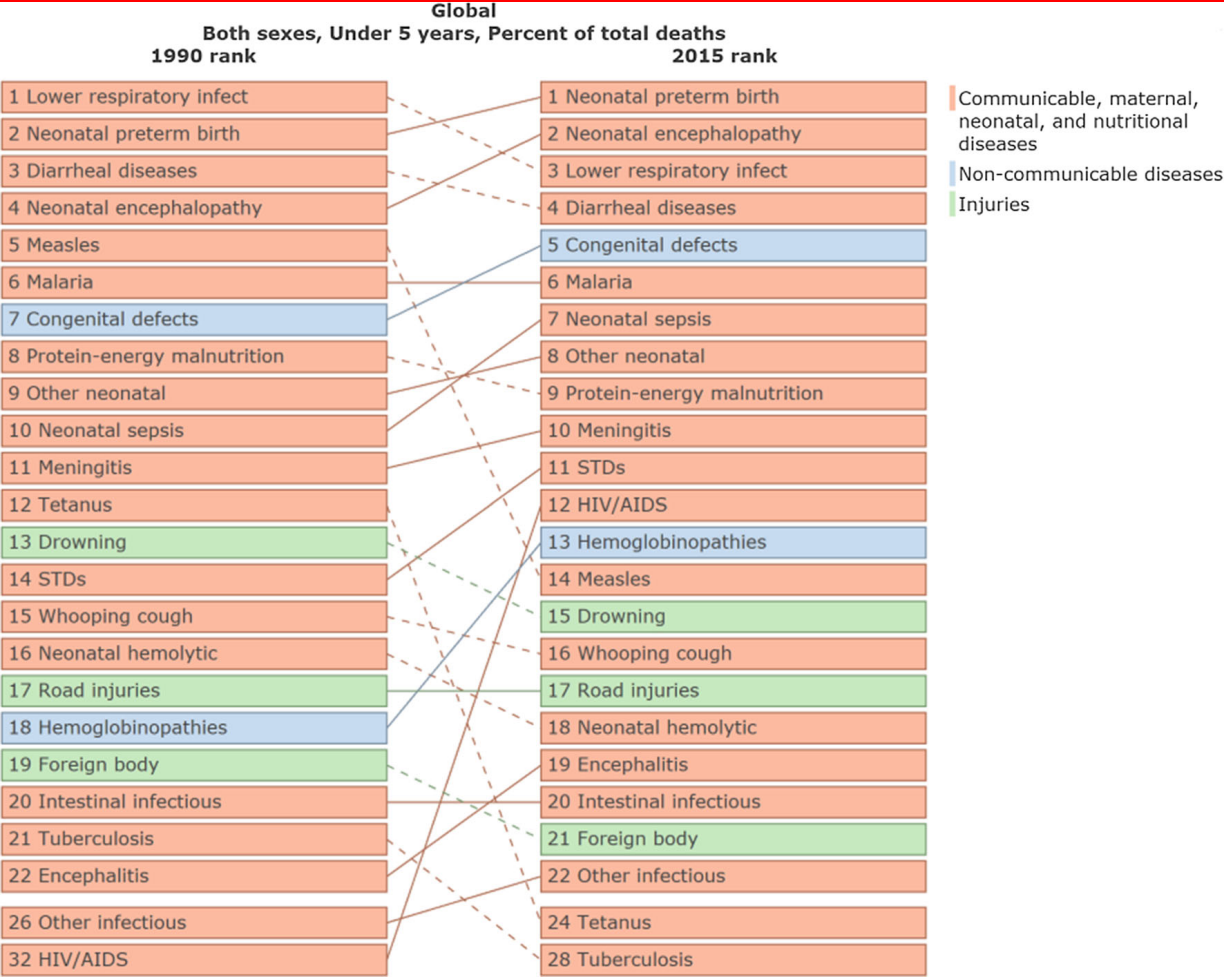
Leading causes of death in children < 5 years of age globally, 1990 to 2015 [14]

During the period between 1990 and 2016, there has been a decline in the number of childhood deaths (less than 5 years of age) from infectious diseases, and an increasing burden from non-communicable diseases, many of which are amenable to surgical care (Fig. 1) [13]. During the same time frame, congenital anomalies increased from the seventh most common cause of death in children under 5 years of age, to the fifth most common (Fig. 2) [14]. This increase places congenital anomalies ahead of HIV, tuberculosis (TB) and malaria as a cause of under 5 mortality. However, efforts to focus the global health agenda on surgical diseases have been largely unsuccessful and have not received the same high-profile funding efforts exemplified by the Global Fund to fight AIDS, TB and malaria and the Gavi Vaccine Alliance [15, 16].

### Children’s surgery is neglected, but essential

The delivery of safe, effective surgical care to children is a critical but neglected area within global surgical efforts. Furthermore, untreated surgical diseases in children may result in lifelong disability [17].

The improvement of essential surgical services will require organization to move the delivery of essential surgical services higher on the agendas of policy leaders and funders [18]. As non-communicable diseases begin to have a larger role within the global health conversation, providers of children’s surgical care must advocate for an increased focus on surgical care for the sick or injured child.

## Designing the process and vision

### Emergence of a coalition

**Fig. 3:**
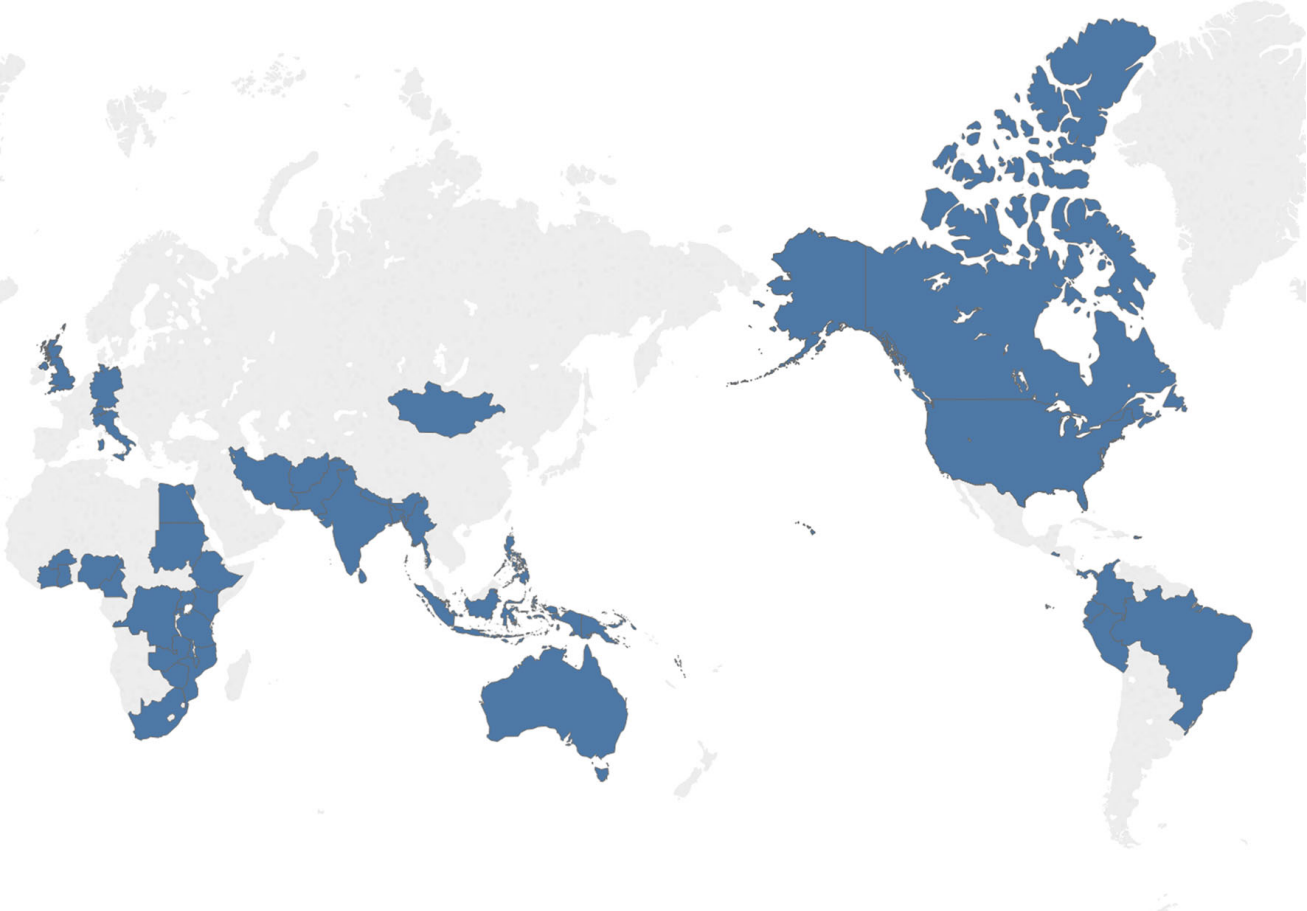
Countries represented at the first, second and third GICS meetings

The Global Initiative for Children’s Surgery (GICS) was established in 2016 to combine the experience and expertise of low- and middle-income country (LMIC) surgical and anesthetic providers with the resources and expertise of high-income country (HIC) partners. GICS was the collective vision of academic pediatric surgeons from Nigeria, Canada, USA and the UK who set out to create a global dialog to advance the access to and quality of safe surgical care for children in low-resource settings. The vision of GICS is that “every child in the world who has a surgical need has access to resources that will optimize his or her surgical care” [19]. The mission of GICS evolved to “define and promote optimal resources for children’s surgery in resource-poor regions of the world by engaging providers of children’s surgical care globally.” With the support of professional associations, including the American Society of Anesthesiologists (ASA), American Pediatric Surgical Association (APSA), the British Association of Paediatric Surgeons (BAPS), the Canadian Association of Paediatric Surgeons (CAPS), the Pan-African Pediatric Surgical Association (PAPSA), the World Federation of Societies of Anaesthesiologists (WFSA), the Canadian Anesthesiologist’s Society (CAS) and International Society for Pediatric Neurosurgery (ISPN), GICS was able to set its agenda and grow into one of the world’s largest coalitions of children’s surgical care providers. GICS currently consists of 266 members from 44 countries (Fig. 3). The collaboration is inclusive of all children’s surgical subspecialties as well as a diverse network of multi-disciplinary care professionals.

To meet their objectives, the founding members of GICS determined “guiding principles,” to ensure that all efforts remained “LMIC centric,” and adaptable to the evolving landscape of global surgery. These values are crucial in differentiating the *ethos* of GICS from other global health initiatives, the terms of which are frequently dictated by HIC leadership rather than by LMIC stakeholders.

### GICS guiding principles

#### “Nothing about us without us” [20]

From the outset, LMIC provider participation and leadership has been prioritized. At the inaugural GICS meeting in May 2016 in London, UK, 52 providers representing 21 countries were in attendance, 27 of whom were providers from 18 LMICs. At the second meeting held in October 2016 in Washington DC, 94 participants (including 43 LMIC providers) representing 76 institutions and organizations from 38 countries (including 30 LMICs) were present. This focus continued at the third meeting in Vellore, India, in January 2018 where 110 participants (two-thirds from LMICs) representing 33 countries were in attendance. The effort to promote LMIC leadership is replicated at every level of the GICS organization with 50% of the Board of Directors being from LMICs and all working groups being led by providers from LMICs.

#### Children’s surgical care: including all specialties

Improvement of surgical care for children in low-resource settings requires the cooperation and input of a wide spectrum of clinical disciplines and allied health professions. A strong emphasis on interdisciplinary collaboration has been a focus of GICS. From the outset, GICS brought together children’s surgeons of all specialties, anesthetists, radiologists and nurses, as well as non-clinical stakeholders (Table 1). In total, there are 13 specialty-based working groups. Each group makes recommendations based on its scope of practice, expected conditions and procedures, as well as infrastructure and equipment required for the practice of its discipline at each facility level.

**Table 1:** Children’s surgical specialties and professionals included within GICS

Children’s surgical specialties	Multi-disciplinary team members	Other stakeholders
Pediatrics and neonatology	Adult general surgeons	World Health Organization
Pediatric surgery	Pediatric surgeons from all surgical specialties	Ministries of Health
Urology	Anesthetists	Charities
Plastic surgery	Critical care specialists	Non-government organizations
Neurosurgery	Emergency physicians	Advocacy experts
Cardiac surgery	Non-physician clinicians	Philanthropists
Orthopedic surgery	Radiologists	
Maxillofacial surgery	Nurses	
Otolaryngology	Biomedical engineers	
Ophthalmology		
Academic surgery		
Research		
Education		
Policy		
Governance		
Management/leadership		

#### Holistic capacity building

The four working groups dedicated to capacity building are mandated to develop strategies to improve the ecosystem of children’s surgery in LMICs. They include: the research, data and quality improvement group, the financing, policy and advocacy group, the training, human resources and education group, and the infrastructure and standards group. The second and third meetings brought together surgeons, anesthesiologists and allied health professionals, representatives from the World Health Organization (WHO), the International Committee of the Red Cross (ICRC), United Nations International Children’s Emergency Fund (UNICEF), Médecins Sans Frontières (MSF), as well as other nonprofit organizations and funding agencies dedicated to addressing the unmet surgical burden in children throughout LMICs.

#### A collaborative character and grassroots movement

GICS deliberately nurtures collaboration by providing equal footing for care providers from diverse backgrounds to contribute their time and ideas toward the achievement of common goals. This is reflected in sponsorships for travel and accommodation for LMIC participants to GICS meetings, made possible through various individual, private, corporate and surgical society donations. Multiple subspecialty organizations supported LMIC providers with whom they had previously established working partnerships. This commitment to maximizing LMIC representation and leadership by active clinical providers fosters improved buy-in from stakeholders and increasing involvement and ownership by LMIC providers. This involvement is fostered not only by the in-person meetings, but also by quarterly online membership meetings.

#### Trainee involvement

Trainees (including surgical residents, registrars and fellows) have been involved in all aspects of the organization from the outset. It has created an avenue for both LMIC and HIC trainees to become involved in the coordination of activities with the GICS leadership, including organization, management and fundraising, all tasks of great importance to individuals seeking careers in global health. The involvement of trainees from countries of all income levels promotes the growth of global health as a career and builds partnerships among the next generation of leaders in children’s surgery.

## Taking action

### Organizational priority setting

Prior to the first GICS meeting in May 2016, delegates from LMICs completed a 21-point pre-meeting survey to identify barriers to providing children’s surgical care in their home countries, as well as potential solutions drawn from personal experience [21]. The delegates highlighted a lack of workforce development as the most important barrier to the provision of children’s surgical care in LMICs. This is consistent with a recent assessment of the state of the pediatric surgery workforce worldwide which demonstrated only 0.26 pediatric general surgeons per million population in Africa compared to 4 per million in the USA—a disparity that is even greater than the numbers suggest given the significantly higher proportion of children in the population of Africa [22, 23].

In addition to the workforce issues, there is a widespread shortage of equipment and resources with 97% of delegates’ home institutions lacking parenteral nutrition for neonates and children, 95% lacking pediatric intensive care and 88% lacking neonatal intensive care. Eighty percent of delegates noted a lack of implants, and endoscopic and laparoscopic equipment.

These deficiencies in workforce and equipment resulted in a list of conditions that providers found particularly difficult to manage, primarily congenital anomalies and tumors. Inadequate funding for children’s surgery was also cited as a barrier; one delegate summarized the problem by saying: “Children are never a priority for politicians, because they are not heard.”

### Working group discussions

Survey results were used to categorize problems into four key areas: infrastructure, service delivery, training and research. These topics were given to working groups consisting of both LMIC and HIC providers to establish steps forward for the organization. The outcomes of these discussions were fed back to the membership at large and discussed to create a consensus on how the organization should address issues facing providers from LMICs (Table 2).

**Table 2:** GICS I consensus meeting outcomes from each working group focus area

Working group focus area	Priorities
Infrastructure	Establish standards for children’s surgical care to be integrated into national surgical plans. Each country should have at least one children’s hospital
Service delivery	Determine conditions that should be managed at each level of healthcare facility. Improve triage and transfer. Determine a standard number of providers per population. Improve prenatal diagnosis, prevention, delivery and rehabilitation
Training	Designate, accredit and support regional training hubs. Standardize training curricula in alignment with professional organizations. Provide scholarships for trainers and trainees and establish training partnerships
Research	Establish regional research centers. Develop a simple, uniform database for quality improvement and research. Prioritize research on birth defects, tumors and pediatric injuries

The providers at the initial meeting felt that sustainable development of safe surgical care for children was dependent on infrastructure investment. LMIC providers also felt that improving surgical infrastructure could not be achieved without integration of children’s health care into national surgical plans. To further this goal, efforts were made to collaborate with child health organizations such as UNICEF and to develop assessment tools to facilitate the inclusion of children’s surgical care into the national surgical plans.

The inability of rural, poverty-stricken patients to access care or avoid suffering unacceptable levels of impoverishing expenditure was the focus area of service delivery. The groups agreed that triage and referral should be prioritized as well as training and support for district hospital teams to manage certain children’s surgical conditions. To facilitate this, standards would need to be set for the appropriate resources and scope of care available at each level of children’s health care.

“Brain drain” was a consistently cited concern, particularly at the trainee surgeon and anesthetist level. There was a consensus that traveling to HICs for training without return incentives is a high-risk strategy. Partnerships between countries of similar healthcare resources or regional partnerships for training were identified as the preferred solution to this issue.

A dearth of research from low-resource settings was identified as a rate-limiting factor for incorporation of children’s surgical care into local, national and global health planning. The establishment of regional research centers to support ethical data collection, grants and research training was identified as a priority. The groups also identified a need for international collaborations around congenital anomalies, oncology and injury including burn care and prevention.

### Implementing the priorities

In response to the positive feedback from the first GICS meeting in London, attention turned to developing solutions for the identified challenges. A second meeting in Washington DC in October 2016 and a third meeting in Vellore, India, in January 2018 were held to allow LMIC and HIC providers from across the globe to meet face to face. Select projects developed by thematic focus (infrastructure, service delivery, training and research) are highlighted below and summarized in Table 3. In accordance with the mission of GICS, all the international projects are collaborations between numerous organizations of all types that were facilitated, in part, through GICS.

**Table 3:** Sample of the current GICS projects by thematic area of focus

Thematic area	Project description	Location
Infrastructure	Partnership between the West African College of Surgeons (WACS) and Harvard Program for Global Surgery and Social Change to integrate children’s surgery into national surgical plans	Nigeria and the WACS region
	‘Kids OR’ charity equipping multiple pediatric operative rooms	East Africa, with plans to expand to LMICs globally
Service delivery	Optimal Resources for Children’s Surgery (OReCS) document describing service delivery stratified by hospital level	Global LMICs
	Global Paediatric Assessment Tool (GAPS) to evaluate existing gaps in pediatric surgical capacity	Global LMICs
Training	Children’s surgical care training program for district hospital adult general surgery teams	India
	Expansion of regional specialty training	Uganda–South Africa
Research	Anorectal Malformation International Database	Global LMICs
	Development of the PaedSurg Africa Research Collaboration with 23 countries participating in a multicenter prospective cohort study of common neonatal and pediatric surgical conditions across sub-Saharan Africa. Current expansion to form the Global PaedSurg Research Collaboration incorporating over 100 countries	Sub-Saharan Africa, expanding to Global LMICs and HICs
	Evaluation of surgery capacity building program in rural Nicaragua	Nicaragua
	Monthly GICS Research Webinars to share project ideas, gain feedback and to enable networking and collaboration	Global LMICs and HICs

### Infrastructure

#### Integrating children’s surgery into national surgical planning

In partnership with the West African College of Surgeons, the Harvard Program of Global Surgery and Social Change, and GICS, Nigeria has begun efforts to incorporate children’s surgery into their national surgical, obstetric, anesthesia and nursing plan.

#### Kids operating room (KidsOR)

KidsOR is a Scottish Registered Charity focused on creating and equipping children’s operating rooms in LMICs. The organization has delivered pediatric operating rooms in Uganda, Tanzania and Malawi. In 2018, KidsOR will deliver new facilities in Rwanda, Mozambique, Malawi, Ethiopia, Uganda and Tanzania. Through the GICS network, the organization is expanding its partnerships further to identify opportunities worldwide [24].

### Service delivery

#### Optimal resources for children’s surgery document

At the second GICS meeting, working groups focused on developing the ‘Optimal Resources for Children’s Surgery’ (OReCS) document detailing standards for children’s surgical care at each level of healthcare facility [25]. The objective of the OReCS document was to inform both national surgical plans in LMICs and provide guidelines for children’s surgery. The American College of Surgeons standards for provision of children’s surgical care served as a model [26]. For each hospital level, GICS members determined which conditions and procedures should be undertaken, the degree of specialty training required, and essential equipment and supplies. LMIC providers felt the development of such a document with input and possible endorsement of groups such as the World Health Organization would provide leverage for resource allocation with local policymakers. Once finalized, the document will be published for wider dissemination.

#### Global assessment of pediatric surgery

In order to reliably measure existing gaps in care and identify urgent needs related to children’s surgery in LMICs, GICS providers created the Global Assessment of Pediatric Surgery (GAPS) tool. GAPS evaluates all healthcare levels and is divided into five sections: human resources, material resources, outcome, accessibility/impact and education. The tool is used to identify potential deficiencies in pediatric surgical care. It serves to inform and align LMIC and HIC partnerships and has been piloted in over a dozen countries.

### Training

#### Partnership between RCS (England), GICS and the Christian Medical College, Vellore, India

The Royal College of Surgeons (RCS) of England has partnered with GICS and the Christian Medical College in Vellore, India, to provide children’s surgical training to rural surgical teams which include adult general surgeons, orthopaedic surgeons, anesthetists and nurses. There is a plan to expand to a wider range of specialties and further locations across India.

#### Facilitation of sub-Saharan Africa to South Africa training collaboration

GICS providers partnered with the Hugh Greenwood Fund from Oxford University to subsidize LMIC providers from sub-Saharan Africa to train in South Africa and other LMIC training centers. Currently, there are three pediatric surgeons, one each from Ghana, Uganda and Zimbabwe, and one pediatric anesthesia trainee from Uganda, training in Johannesburg.

### Research

#### PaedSurg Africa research collaboration

The PaedSurg Africa Research Collaboration has been established incorporating 220 children’s surgical care providers from 23 countries across sub-Saharan Africa into a unified research network. Many of these collaborators were invited to participate at the GICS meetings. These collaborators have undertaken the largest multicenter prospective cohort study of children’s surgery in this region to characterize the current management and outcomes of common children’s surgical diseases [27]. Participation has created opportunities for local, national and international presentations and networking. This collaboration is currently being expanded to form the ‘Global PaedSurg Research Collaboration’ with the first study focusing on the management and outcomes of congenital anomalies [28].

#### Anorectal malformation registry

An anorectal malformation (ARM) database is being prospectively maintained over a 1-year period by pediatric surgeons and allied health professionals in low-resource settings. This effort, facilitated and supported by GICS, fills a vital gap as the majority of international registries currently collecting long-term data on congenital anomalies are based in HICs, with very few covering LMICs, particularly sub-Saharan Africa [29, 30].

#### Monitoring and evaluation in Nicaragua

GICS is involved in a partnership project with Operacion Sonrisa Nicaragua and the Nicaraguan Ministry of Health. The goal of this collaboration is to improve access to surgical care by bringing safe surgical care for adults and children closer to the most vulnerable communities in rural Nicaragua. Following a baseline assessment of surgical capacity, Nicaragua has begun investment in training, infrastructure and surgical equipment. GICS is assisting with monitoring and program evaluation [8, 31].

## Determining future directions

Moving forward GICS will continue to provide a forum to support new collaborations, between HIC and LMIC providers, but especially between LMIC providers. The future directions of the organization will be set by the LMIC members. The formalization of GICS into a 501(c)3 allows the organization to remain flexible and responsive to the needs of its members while ensuring that the day-to-day actions of the organization reflect its founding principles. Membership is defined not by financial contribution, but by participation in projects and meetings. As GICS continues to evolve, we acknowledge the challenges ahead. These include maintaining sustainability in the absence of dedicated funding, operating alongside disparate global surgical initiatives and partnering when there is mutual benefit while preserving LMIC leadership. Despite these challenges, GICS remains dedicated to ensuring that the surgical and perioperative needs of children on the global scale are heard and addressed.

## Conclusions

Though the field of global surgery is expanding rapidly, significant gaps exist in children’s surgical care. These include access to surgical care, metrics for measuring children’s surgical capacity and outcomes, and the absence of children’s surgery in national health plans. GICS is well positioned to help address these challenges. By promoting close collaboration among children’s surgical care providers worldwide and providing a platform for joint training, research and advocacy, GICS seeks to support all providers in their mission to improve surgical care for children.

We believe that the structure of GICS and its guiding values is critical to the accomplishment of its grand vision: That “every child in the world who has a surgical need will have access to resources that will optimize his or her individual care.”
